# Characterization of *Omics* Components in Human Milk: A Systematic Review

**DOI:** 10.34763/jmotherandchild.20252901.d-24-00044

**Published:** 2025-09-12

**Authors:** Julián Manuel Espitia Angel, Sergio Agudelo-Pérez, Laura Manuela Olarte Bermúdez, Daniela Del Pilar Chaparro Rojas, Sandy Daniela Bonilla Herrera, Mariana Gómez Merchán

**Affiliations:** Department of Pediatrics, School of Medicine, Universidad de La Sabana, Chía, Cundinamarca, Colombia; Deparment of Pediatrics, School of Medicine, Universidad de La Sabana. Campus Puente del Común, Km 7, Autopista Norte de Bogotá, Chía, Cundinamarca, Colombia

**Keywords:** Human Milk Omics, Breastfeeding and Infant Nutrition, Maternal Health and Lactation Stages, Bioactive Molecules and Precision Nutrition

## Abstract

**Background/Aims:**

The proteome, lipidome, glycome, and metabolome of human milk are critical for newborn nutrition and health, and offer personalised, non-pharmacological interventions. This systematic review aims to characterise the omics components of human milk according to maternal health and lactation phases, summarising current knowledge based on high-resolution analytical techniques.

**Methods:**

We conducted a systematic review according to the PRISMA 2020 guidelines. The search was performed between August and September 2022 using Medline, EMBASE, Scopus, LILACS, and Web of Science. Observational studies that analysed human milk at any lactation stage using mass spectrometry or nuclear magnetic resonance to characterise nutrients, biomolecules, or bioactive compounds were included. In total, 55 full-text articles were included in this study.

**Results:**

Glycomics is the most frequently studied omics, followed by proteomics, metabolomics, and lipidomics. Analyses revealed that maternal comorbidities and lactation phases influence the composition of human milk. Fucosylated HMOs showed a protective role against infectious diseases, while elevated levels of protease inhibitors were found in milk from allergic mothers and elevated immunoglobulins were present in milk from mothers with COVID-19. Endocannabinoid profile is associated with improved neonatal sucking ability, while fatty acid-derived metabolites are correlated with infant growth. Distinct omics patterns have also been identified in mothers with diabetes, hypothyroidism, and obesity.

**Conclusion:**

Understanding the omics profile of human milk can guide precise nutrition and improve human milk substitutes. Further research integrating omics data with maternal and infant outcomes will be essential to advance knowledge and support infant health.

## Introduction

Breast milk is the optimal source of nutrition for infants, recommended exclusively during the first six months of life, and continued alongside complementary feeding until at least two years of age [1]. It confers critical benefits in nutrition, metabolism, neurodevelopment, and immune protection, both in the short and long terms. These advantages are derived from breast milk’s intricate composition of macronutrients, micronutrients, and bioactive molecules, which interact dynamically with infants to support essential biological processes and adapt to individual needs and conditions [2]. Notably, the composition of breast milk varies among individuals and is adjusted in response to maternal health status and environmental factors [3,4,5,6,7].

Recent advancements in high-resolution analytical technologies, such as mass spectrometry (MS), nuclear magnetic resonance (NMR), and chromatographic techniques (GC and LC), along with advances in bioinformatics and statistical approaches, have markedly enhanced the ability to analyse complex biological matrices. These innovations have given rise to the field of *omics* and enabled comprehensive characterization of the proteome, lipidome, glycome, and metabolome in biological fluids, including human milk [8, 9]. *Omics* technologies have elucidated the metabolic profile of human milk, its temporal variations, and its remarkable capacity to adapt to diverse maternal and neonatal conditions [10]. This growing body of knowledge highlights the central role of human milk in newborn nutrition and health, offering personalised non-pharmacological interventions for both healthy and critically ill infants [1, 7].

In this context, we conducted a systematic review of the literature to characterise human milk from an *omics* perspective, integrating evidence from high-resolution analytical technologies across different lactation phases with clinical and biological observations of the mother-infant dyad.

## Methodology

We conducted a systematic review of the literature following the PRISMA 2020 (Preferred Reporting Items for Systematic Reviews and Meta-Analyses) guidelines [11].

### Literature search strategy

A literature search was performed between August and September 2022 across the electronic databases MEDLINE (via PubMed); EMBASE; Scopus; Latin American and Caribbean Health Sciences Literature (LILACS); and Web of Science. Additionally, a snowball sampling approach was employed to screen the reference lists of eligible full-text articles and identify further relevant studies. The search was limited to publications in English and Spanish, and observational studies (cohort, case-control, and cross-sectional) were eligible for inclusion.

The search strategy applied in MEDLINE was as follows, and was adapted accordingly for the other databases: ((“Milk, Human” OR “Breast Milk Expression” OR “Lactation” OR “Breast Feeding” OR “Colostrum”) AND (“Mass Spectrometry” OR “Proton Magnetic Resonance Spectroscopy”)) AND (“Bioinformatics” OR “Proteomics” OR “Metabolomics” OR “Lipidomics” OR “Oligosaccharides” OR “Glycomics”).

### Study eligibility criteria

- Analysed human milk at any stage of lactation (colostrum, transitional, or mature milk).
- Employed mass spectrometry (MS) and/or nuclear magnetic resonance (NMR) as analytical platforms, and
- Characterised nutrients, biomolecules, and/or bioactive compounds using *omics* approaches.

The following exclusion criteria were applied:

- Literature reviews (narrative, systematic, scoping, etc.), conference proceedings, poster abstracts, correspondence to the editor, and unpublished data.
- *In vitro* studies, and
- Studies characterising the *omics* profile of donor milk in milk banks and/or pasteurised human milk.

### Screening and inclusion of studies

All retrieved records were independently screened and assessed for eligibility by two reviewers in a blinded manner. Disagreements were resolved through consensus. The Bioinformatics Systematic Literature Review (BiSLR) instrument was used to guide decision-making regarding study inclusion [12]. The initial screening of titles and abstracts was performed using the Rayyan® web application, which also allowed for the identification and removal of duplicate records across databases. The full texts of potentially relevant articles were retrieved and independently reviewed in detail by both reviewers.

### Data extraction and synthesis

For each included study, we extracted data on the journal name; first author; publication year; country; clinical characteristics of the mothers; *omics* domain (proteomics, metabolomics, glycomics, lipidomics); analytical technology employed; and bioinformatics database used.

The extracted data were qualitatively synthesized and organised into tables, categorising the findings by *omics* domain, maternal health status, and lactation phase to facilitate comparisons and highlight patterns across studies.

## Results

**Figure 1.**
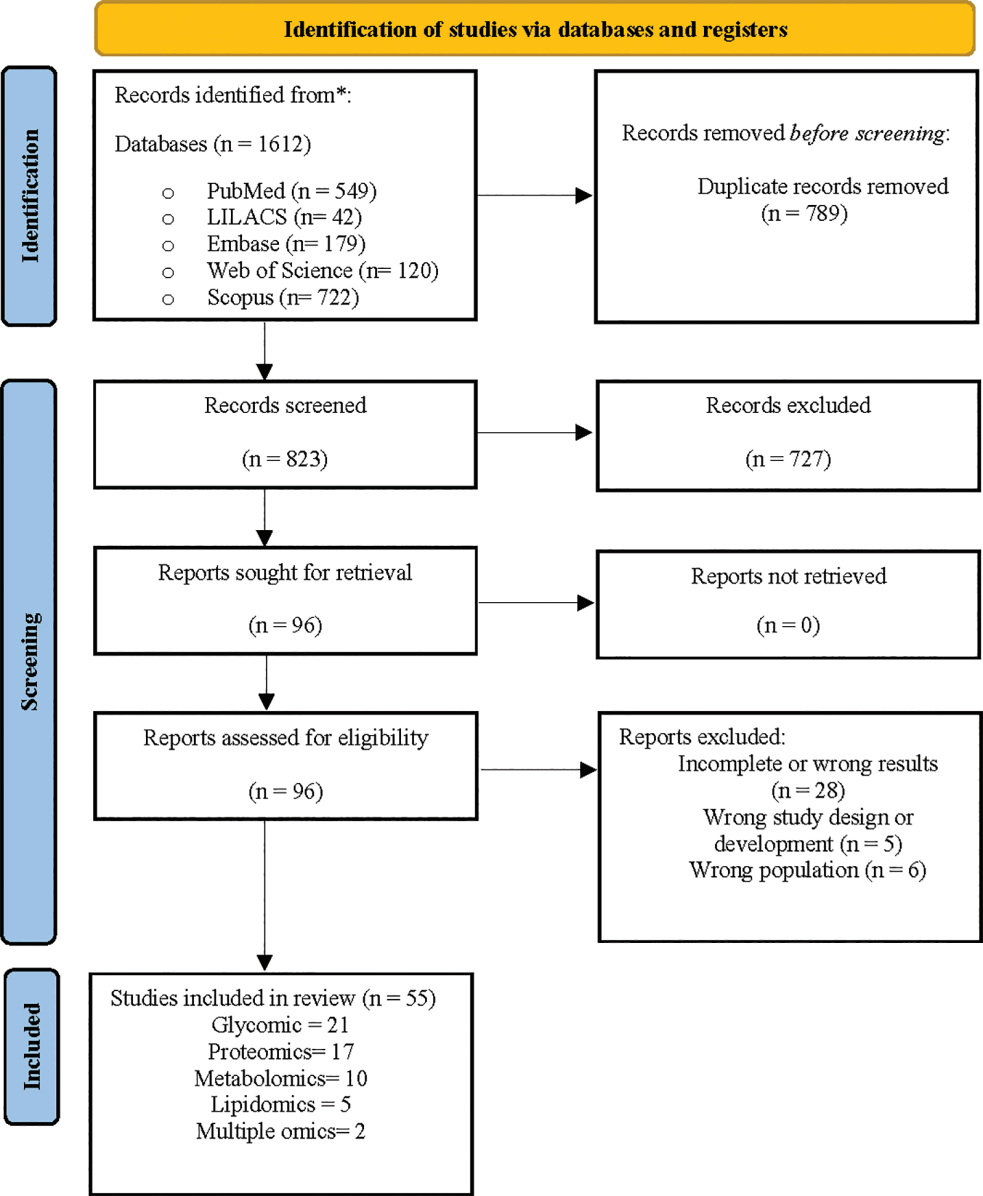
Flow chart study.

The study selection process (Figure 1) began with 1,612 records, which were reduced to 823 after removing duplicates. Following title and abstract screening, 96 full-text articles were reviewed, and 55 met the inclusion criteria, including 21 on glycomics, 17 on proteomics, 10 on metabolomics, 5 on lipidomics, and 2 on multi-omics. Bibliometric details and findings are presented in Supplementary Table 1, while Table 1 summarizes the key results by *omics* component, maternal health status, and lactation phase. Healthy mothers were the subject of 82% of the studies; the rest included those with gestational diabetes (n = 4), allergies (n = 2), obesity (n = 1), iodine deficiency (n = 1), hypothyroidism (n = 1), or COVID-19 (n = 1). Regarding lactation phase, 44% analysed colostrum, 42% analysed transitional, 82% analysed mature, and 25% analysed all phases.

**Table 1.** Summary of the main findings organized by omics component, maternal health status, and lactation phase.

**Omics**	**Maternal health: healthy**	**Maternal health: with comorbidities**	**Lactation phase: colostrum**	**Lactation phase: transitional**	**Lactation phase: mature**
Glycomics	High diversity of HMOs; ↑ fucosylation early; ↓ over time; neutral and sialylated HMOs decrease progressively	↑ DFpLNnH in allergic mothers; differences in secretor/non-secretor patterns; ethnic variation	High HMO concentrations, fucosylated and sialylated; immune protection	HMOs decrease, shifting patterns (e.g., LNT, LNFP)	3′-FL increases over time; ↓ total HMOs
Proteomics	High levels of immune proteins (lactoferrin, IgA, etc.); proteins supporting development and metabolism	↑ protease inhibitors in allergic mothers; ↑ apolipoproteins in diabetes; ↓ IgA in diabetes; ↑ immune proteins in COVID-19	High diversity of low-molecular-weight and immune proteins	Decline of early proteins; stabilization	Predominance of growth and metabolism-related proteins
Metabolomics	Amino acids, essential fatty acids, endocannabinoids support growth and development	↑ lipids in obesity; alterations in diabetes, hypothyroidism, and iodine deficiency	↑ carbohydrates, amino acids, essential fatty acids	Gradual decline of initial lipids and metabolites	↑ vitamins D3, D2, biotin; ↓ cholesterol, tocopherols
Lipidomics	High lipid diversity: very long-chain fatty acids identified; ↑ saturated and unsaturated fatty acids	Minor variations reported	High in phosphatidylcholine, triglycerides, ether-phospholipids	Decrease in plasmalogens and polyunsaturated lipids	Stabilized lipid profile; predominance of lipids supporting microbiota and development

* Abbreviations: HMO: Human Milk Oligosaccharides; DFpLNnH: difucosyl-para-lacto-N-neohexaose; LNT, lacto-N-tetraose; LNFP: lacto-N-fucopentaose; 3′-FL, 3′-fucosyllactose; IgA: immunoglobulin A; COVID-19: coronavirus disease 2019; D3: vitamin D3; D2: vitamin D2.

## Glycomics

### Oligosaccharides (HMO) profile of the milk of healthy mothers

The composition of HMOs in milk from healthy mothers is influenced by maternal and neonatal factors, including age, allergic history, BMI during pregnancy, parity, delivery mode, gestational age, and infant sex [13,14]. Genetic determinants, such as the *Se* and *Le* genes on chromosome 19, play key roles, with *Se* associated with higher 2′-fucosyllactose (2′FL) levels and *Le* with higher lacto-N-tetraose (LNT) and lacto-N-fucopentaose II (LNFP II) [15, 16].

High-resolution analyses have detected elevated levels of 2′-FL, α1, 2-fucosylated HMOs, and bifidobacteria-associated oligosaccharides [13, 17, 18]. HMO concentrations were not significantly related to growth in the first four months but were lower in infants with excessive weight gain [14, 19]. Geographic differences were observed, with Latino mothers exhibiting a higher prevalence of secretory HMO patterns [16].

### HMO profile of the milk of mothers with comorbidities

Higher levels of difucosyl-para-lacto-N-neohexaose (DFpLNnH) have been reported in Brazilian mothers with allergic diseases, especially in mothers with the *Se+* phenotype [14]. Variability in the HMO composition was noted among the *SeLe* groups was associated with maternal allergic conditions [14].

### HMO profile of human milk in the lactation phases

The HMO concentrations and composition evolve markedly across lactation stages [18, 20,21,22,23,24]. Chaturvedi et al. documented a decline in the total HMO concentration from approximately 9 g/L in colostrum to approximately 4 g/L at one year postpartum, with a specific decrease in α1,2-fucosylated HMOs [18, 20,21,22,23,24]. On the other hand, 3′-fucosyllactose (3′-FL) increased significantly during this period, reaching up to four times its initial concentration in one year [13, 18, 20].

This transition is characterised by a shift from neutral, non-fucosylated HMOs to a higher proportion of fucosylated species, while neutral HMOs declined more rapidly, particularly during the transition to mature milk [22]. In colostrum, high levels of fucosylated HMOs persist during the first four weeks to support the infant’s immature immune system and intestinal barrier [13, 20, 25]. Transitional milk was enriched in lacto-N-fucopentaose III, lacto-N-hexaose, monofucosyllacto-N-hexaose I and III, and acidic HMOs [13, 26]. Wang et al. further observed that mature milk in the first month of lactation contains higher concentrations of the isomer 1-fucosyl-paralacto-N-hexaose III and difucosyllacto-N-hexane-b, whereas 3′-peaks around six months postpartum [13].

## Proteomics

### Proteomic profile in the milk of healthy nursing mothers

Human milk has a complex protein composition; its key components include serum albumin, lactalbumin, caseins, lysozyme, lactotransferrin, bile salt-activated lipase, and glycoproteins such as polymeric Ig receptor, lactoferrin, and mannose receptor 1 [27,28,29]. Other proteins detected included bovine caseins and β-lactoglobulin, which may have been derived from maternal diet [30]. Furthermore, specific amino acids and metabolites, such as arginine, tyrosine, hydroxybutyrate, niacinamide, choline, and lacto-N-fucopentaose, have been identified as potential predictors of weight gain in preterm infants during hospitalization [19].

Human milk is rich in immune-related proteins and calcium-regulating proteins. Lactoadherin provides protection against rotavirus, while lactoferrin, adipophilin, and butyrophilin exert antibacterial effects through iron sequestration [31]. Peptides derived from lactoferrin promote growth and antimicrobial properties [32]. Novel proteins, including polymeric immunoglobulin receptors and human leukocyte antigens (HLA), facilitate IgA antibody transport [33]. FAM20A and other calcium-associated proteins play critical roles in regulating calcium homeostasis and preventing pathological calcification of mammary epithelial cells during lactation [29].

### Proteomic profile in human milk according to maternal comorbidities

The protein composition of human milk is influenced by maternal health status. In mothers with allergic diseases, the levels of protease inhibitors, cystatin C, and apolipoproteins are elevated — potentially limiting allergen penetration through the epithelium — while transthyretin and calcium-binding protein A1 are reduced [34]. In those with gestational diabetes mellitus, higher levels of Apolipoprotein A1 and lactoferrin-related proteins have been reported, along with reduced IgA concentrations [35, 36]. In mothers with hypothyroidism, immune-related proteins increased, but proteins associated with energy metabolism and cytoskeletal integrity, such as actin, tubulin, and GAPDH, decreased [37].

During the COVID-19 pandemic, colostrum from infected mothers exhibited increased levels of immune proteins, including SIgA1 and complement factors, along with a marked reduction in casein [38].

### Proteome profile of human milk according to the stages of lactation

High-resolution analyses have revealed substantial changes in the proteome during lactation. Colostrum is characterised by an abundance of low-molecular-weight proteins and peptides, which decline rapidly within the first month postpartum and stabilize in mature milk approximately 60 d after birth [39]. Early lactation also features elevated levels of essential and branched-chain amino acids such as leucine, isoleucine, and valine [40]. Proteins such as R-1-antitrypsin, carbonic anhydrase, and E-cadherin predominate in the early stages, whereas fatty acid-binding proteins and lysozyme C become more prevalent after six months [41]. Additionally, tryptophan, histidine, and peptides associated with purine and pyrimidine nucleotide metabolism increase throughout lactation, reflecting the increasing requirements for DNA synthesis and cell proliferation [42].

## Metabolomics

### Metabolome profile in human milk from healthy nursing mothers

Human milk from mothers of preterm infants exhibits higher concentrations of carbohydrates — particularly lactose — and lipids, such as oleic and linoleic acids [43]. Alexandre-Gouabau et al. identified a metabolic profile associated with faster growth in preterm infants that was characterised by elevated levels of branched-chain amino acids, insulinotropic factors, lacto-N-fucopentaose, choline, hydroxybutyrate, tyrosine, and arginine [19]. Conversely, the profile linked to slower growth included higher levels of linoleic acid and reduced levels of purine nucleosides and glutamate/glutathione metabolites [19]. Gaitán et al. reported an endocannabinoid metabolome in human milk that included arachidonic acid (ARA), oleoylethanolamide (OEA), and palmitoyl glycerol (PG), suggesting that these metabolites may enhance neonatal sucking and feeding capacity [44].

### Metabolomic profile in human milk from mothers with comorbidities

In mothers with a high body mass index (BMI), human milk contains elevated concentrations of glycerophospholipids and sphingomyelins, which can potentially increase the risk of childhood allergies [45]. According to Isganaitis et al., milk from obese mothers showed higher levels of insulin, leptin, TNF-α, and IL-6, and metabolites such as adenine and 5-methylthioadenosine were positively correlated with both maternal BMI and infant adiposity [46]. Similarly, Yue et al. identified metabolomic differences in the milk of mothers with diabetes mellitus, including altered levels of d-(+)-glucose, paraxanthine, theobromine, and theophylline, along with reduced concentrations of glycerol-2-phosphate and glycerol-3-phosphate [47]. Additionally, milk from iodine-deficient mothers exhibited significant alterations in 31 metabolites, including reduced levels of selenium, zinc, and copper [48].

### Profile of the metabolome of human milk in the phases of lactation

The metabolome of human milk undergoes substantial changes during lactation. In colostrum, lower levels of glutaric acid and lysine were observed than those in mature milk, which showed increased concentrations of these metabolites [49]. Prostaglandin E2 (PGE2), derived from arachidonic acid, is initially low in colostrum but increases in mature milk, supporting intestinal repair and immune tolerance in the neonate [49]. Other components, including lipids, phospholipids, α-tocopherol, cholesterol, cholesterol esters, fucose, and d-glucosamine acid, decline over time [50]. Conversely, vitamin D3, D2, and biotin levels rise throughout lactation, whereas retinoyl-β-glucuronide and γ-tocopherol levels progressively decrease [42].

## Lipidomics

### Lipidomic profile of milk from healthy nursing mothers

Studies on the lipid composition of human milk from both full-term and preterm mothers, have identified up to 235 distinct lipids belonging to 16 subclasses, with significant differences in colostrum lipids depending on gestational age [51, 52]. Previously unreported very-long-chain fatty acids (C26:0 and C26:1) were also detected [53]. In preterm infants, the lipid profile shows elevated levels of phosphatidylethanolamine and phosphatidylcholine compared to term milk, while diacylglycerol and ceramide levels are reduced [51]. These lipidomic differences are associated with metabolic pathways, neurodevelopment, and LXR/RXR signalling in neonates [51].

Fatty acid-derived metabolites have also been linked to growth outcome. Faster infant growth correlates with higher levels of tyrosine, arginine, medium-chain saturated fatty acids (e.g., pentadecanoic and myristic acid), triglycerides, phosphatidylcholine, and sphingomyelin [19]. Conversely, slower growth is associated with higher concentrations of plasmalogen-derived oleic acid, arachidonic acid, ceramide, and very-long-chain fatty acids [19].

### Lipidomic profile in human milk during the lactation phases

The lipid profile of human milk undergoes significant changes during lactation [54]. Early lactation is characterised by elevated concentrations of odd-chain fatty acids, triglycerides, ether glycerophospholipids, and long-chain polyunsaturated fatty acids (LC-PUFAs). As lactation progresses, plasmalogens and polyunsaturated phospholipids decrease. During the first 15 d postpartum, phosphatidylcholines and phosphatidylglycerols decrease, while lysophosphatidylethanolamines and lysophosphatidylcholines increase [52, 54, 55].

In addition, cholesterol and cholesterol ester levels decline between day 2 and day 84 post-partum [50]. Mature milk is characterised by higher levels of saturated and unsaturated fatty acids, which play a crucial role in supporting infant development and establishing early gut microbiota [40].

## Discussion

This systematic review describes the *omics* components of human milk by integrating evidence from healthy mothers, as well as mothers with comorbidities, at different lactation stages. The findings were systematically categorised by maternal health status and lactation phase, underscoring the dynamic and adaptive nature of milk composition. By combining glycomics, proteomics, metabolomics, and lipidomics, this review highlights how high-resolution analytical platforms have advanced our understanding of human milk as a complex bioactive fluid. The observed variations in bioactive molecules and macronutrients across maternal and lactation stages reveal the potential mechanisms by which milk may provide personalised support for infant nutrition, immune protection, microbiome development, and growth.

### Carbohydrates in human milk

The data revealed substantial variability in the types and amounts of HMOs, with by maternal genetic factors (e.g., FUT2 and FUT3 gene status), health, environmental and geographic context, and lactation phase all having effects [56]. Over time, there is a consistent increase in 3′-fucosyllactose (3′-FL) and overall fucosylation, while sialylation and non-fucosylated neutral oligosaccharides progressively decrease. This dynamic adaptation reflects the evolving needs of the infant’s immune system and microbiota maturation during lactation. Functionally, 3′-FL inhibits pathogen adhesion to epithelial cells, contributing to mucosal protection [14]. Sialylated HMOs, including 3′-sialyllactose (3′-SL) and 6′-sialyllactose (6′-SL), show antiviral activity against influenza and possibly other respiratory viruses [57]. Milk rich in 1,2-fucosylated oligosaccharides also reduces the risk of gastrointestinal infections by pathogens, such as *Escherichia coli*, *Campylobacter jejuni*, and noroviruses, by competitively inhibiting their gut receptor binding [58]. Lower levels of lacto-N-difucohexaose I (LDFH-I), which is closely associated with 2′-fucosyllactose (2′-FL), correlate with higher rates of calicivirus-associated diarrhoea, underscoring the protective role of 2′-FL in blocking mucosal pathogen-host interactions [58]. Beyond their antimicrobial and antiviral properties, HMOs act as prebiotics, promoting colonization by beneficial *Bifidobacterium* species and enhancing gut barrier function and immune modulation. These findings illustrate the critical contribution of milk oligosaccharides to infant health beyond nutrition, positioning HMOs as key components of personalised protection in early life.

### Proteomics in human milk

Colostrum from SARS-CoV-2-infected mothers contains higher levels of immunological proteins, such as IgA and complement components, and lower levels of ribosomal proteins [38]. Secretory IgA (SIgA) not only neutralizes viral particles, but also modulates mucosal immune responses by transporting antigens to dendritic cells, thus fostering immune education in infants [59]. The observed reduction in ribosomal proteins in colostrum may represent an adaptive response to inhibit viral replication, as ribosomal machinery is essential for viral protein synthesis [60]. These findings underscore the dynamic immune adaptation of breast milk during maternal infections, providing tailored protection for neonates.

In addition to infections, proteomic analyses of human milk have revealed associations with neonatal growth and development. Alexandre-Gouabau et al. identified specific proteins and metabolites in milk that could serve as biomarkers to predict weight gain in preterm infants during hospitalization [19, 61]. This supports the growing concept of precision nutrition, in which milk composition is leveraged to optimize individualized nutritional support for vulnerable neonates.

Additionally, evidence suggests that human milk may contribute to allergy prevention and immune regulation in the offspring. Studies have shown that milk from mothers with allergic conditions (e.g., asthma, rhinitis, and eczema) contains elevated levels of protease inhibitors, as well as immunomodulatory proteins such as cystatin C and apolipoproteins [34]. These molecules may help maintain epithelial barrier integrity, regulate protease-mediated inflammation, and modulate immune sensitivity, thereby potentially conferring on children protection against allergic diseases.

### Metabolomics in human milk

Colostrum is metabolically rich and contains elevated concentrations of essential and branched-chain amino acids that support immune function, growth, and neurodevelopment in infants [49]. Amino acids such as leucine, isoleucine, and valine are critical for neonatal protein synthesis, energy metabolism, and signalling pathways involved in cell proliferation and differentiation. In addition, long-chain fatty acids such as oleic and linoleic acids are abundant in early milk and contribute to gut microbiota maturation, epithelial integrity, and anti-inflammatory pathways in early life [40].

The metabolome of human milk is dynamic and reflects both the maternal status and lactation stage. Variations in the metabolite profiles during lactation can modulate infant health, growth, and developmental outcomes. For instance, in iodine-deficient mothers, approximately 31 metabolites show altered expression, with disruptions in lipid metabolism, glycolysis, and amino acid cycling pathways [48]. These metabolic alterations can impair neonatal neurodevelopment and growth, underscoring the need for adequate maternal nutrition during lactation.

Maternal obesity significantly influences the metabolomic composition of milk. Breast milk from obese mothers is often characterised by higher levels of pro-inflammatory markers, glycerophospholipids, sphingomyelins, and monosaccharides, along with lower levels of protective bioactive factors [62]. These alterations are associated with increased infant adiposity, as well as a heightened risk of childhood obesity and metabolic syndromes. Specific metabolites, such as iditol, galactitol, sorbitol, N-methyl-d-aspartic acid, L-serine, and D-glutamic acid, have been implicated in excessive neonatal growth and fat accumulation [63].

These findings suggest that the metabolome of human milk not only provides essential building blocks for infant development, but also reflects maternal health and environmental exposures, offering opportunities to identify biomarkers for monitoring maternal-infant health and tailoring nutritional interventions.

### Lipidomics in breast milk

Recent studies have revealed a distinctive endocannabinoid profile in human milk that appears to contribute to improved motor function and coordination of sucking and swallowing in neonates [44]. Given that early weaning is often attributed to sucking difficulties and perceived insufficient milk supply, it would be valuable to further investigate whether this endocannabinoid profile could serve as a biomarker for the risk of early weaning or feeding difficulties [64, 65].

In mothers of pre-term infants, human milk shows significantly elevated levels of phosphatidylcholine [66]. This lipid is a critical precursor of choline, which is essential for the structural and functional development of the central nervous system. Preterm infants have increased choline requirements and insufficient choline intake, which can adversely affect neurocognitive development and pulmonary maturation [67]. Furthermore, alterations in bioactive lipids like plasmalogens has been linked to neonatal complications, including bronchopulmonary dysplasia [68], as well as to long-term conditions, including chronic obstructive pulmonary disease [69]; Alzheimer’s disease [70]; Parkinson’s disease; and Down syndrome [71]. These associations underscore the potential of lipidomics as a tool for identifying at-risk infants and for informing targeted nutritional interventions.

These findings underscore the complexity and adaptability of human milk, which transcends nutrition to act as a dynamic bioactive fluid tailored to the infant’s stage, health, and maternal condition. Multi-*omics* approaches—glycomics, proteomics, metabolomics, and lipidomics—have revealed molecular networks that nourish, modulate immunity, shape microbiota, support neurodevelopment, and protect against disease. This system-level perspective positions human milk as a model for personaliszed medicine, offering insights into preventive and therapeutic strategies beyond infant nutrition. Future research combining *omics*, metagenomics, and longitudinal outcomes will be essential to fully elucidate these mechanisms and inform interventions to improve maternal and infant health globally.

This review had several limitations. Studies on the metagenomic profile of human milk—a key component for understanding the milk microbiota and its impact on infant health—were not included. Heterogeneity in study designs, analytical platforms, and reporting limits quantitative synthesis and comparability. Most of the studies were observational and cross-sectional, restricting causal inferences. Finally, restricting English and Spanish may have excluded relevant studies.

Among its strengths, this is the first study to integrate findings from four major *omics* disciplines, systematically summarizing the molecular components of human milk as they fluctuate with maternal health and lactation stage. A rigorous search strategy, adherence to PRISMA guidelines, and clinically relevant categorization of findings provide robust synthesis and highlight knowledge gaps, making it a valuable resource for clinicians, researchers, and policymakers.

## Conclusion

Advances in high-resolution *omics* and bioinformatics have revealed the dynamic and individualised composition of human milk, shaped by maternal genetics, health, and lactation stages. Beyond HMOs, which are key for immunity, microbiota, and disease prevention, proteomics, metabolomics, and lipidomic studies highlight bioactive molecules that support growth, neurodevelopment, and immune regulation. Future research should clarify causal links with infant outcomes and how maternal factors influence the “nutriome.” Deciphering this network reinforces the unique role of milk and provides information for precision nutrition, neonatal care, and improved human milk substitutes.

### Key Points

- Carbohydrates in breast milk, particularly HMOs, contribute to the protection against infectious and allergic diseases through microbiota modulation and pathogen inhibition.
- *Omic*-derived components of human milk vary significantly depending on maternal comorbidities and lactation phases, reflecting personalised adaptation.
- An endocannabinoid profile unique to breast milk may play a role in infant motor function, sucking, and swallowing skills, with implications for early weaning risk.
- Specific metabolite profiles in human milk are associated with infant growth trajectories and may serve as biomarkers to guide nutritional intervention.
- Distinct *omics* patterns have been observed in the milk of mothers with diabetes mellitus, hypothyroidism, and obesity, potentially influencing infant development and health outcomes.

## Supplementary materials

**Table S1.** Characteristics of the studies included in the systematic review: bibliometric data, omics component analyzed, analytical platform, bioinformatics database, clinical characteristics, and main findings.

**Bibliometric data (author, year, country and reference)**	**Omics**	**Method of technology**	**Bioinformatics base**	**Clinical characteristics. (lactation phase, healthy or sick).**	**Results**
Wieruszeski J et al., 1985, France	Glycomics	FAB-MS and LC-MS.	Not reported	Not reported	Identified two novel oligosaccharides (lacto-N-sialylated fucopentaose I and II), likely reflecting mammary glycosyltransferase activity during lactation.
Grönberg G et al., 1990, Sweden	Glycomics	SEC-IEC-HPLC-NMR.	Not reported	Not reported	Identified three novel disialylated oligosaccharides in human milk.
Finke B et al., 1999, Germany	Glycomics	MALDI-MS.	Not reported	Not reported	Detected acidic and neutral oligosaccharides, including complex neutral structures of up to 35 monosaccharide units.
Chaturvedi P et al., 2001, USA	Glycomics	rpHPLC.	Not reported	Transitional and mature milk from healthy mothers	Oligosaccharides varied in quality and quantity during lactation, with higher fucosylated and sialylated forms early on, decreasing after 26 weeks postpartum; low concentrations may inhibit pathogen binding.
Niñonuevo M et al., 2008, USA	Glycomics	HPLC-Chip/TOF-MS.	Not reported	Colostrum, transitional and mature milk from healthy mothers	LNT, LNnT and LNFP I/V were most abundant and stable throughout lactation, with higher fucosylated vs. sialylated HMOs from day 1 to 71, potentially influenced by maternal or infant factors.
Albrecht S et al., 2010, Netherlands	Glycomics	Off-line CE-LIF, Online CE-LIF-ESI-MS.	Not reported	Not reported	Detected major peaks of SL, DS-LNT, S-LNT, FL, LNDFH, LNT, LNFP I/III, LNFP II, and DF-L.
Dallas D et al., 2011, USA	Glycomics	HPLC-Chip/TOF-MS	In-house Postgres relational database	Mature milk in healthy mothers.	Identified 52 N-linked glycans (84% fucosylated, 47% sialylated); breast milk provides Neu5Ac, important for neonatal brain development.
Xu G et al., 2017, USA	Glycomics	HPLC y UPLC/QqQ-MS.	Not reported	Transitional and mature milk from healthy mothers	Most oligosaccharides decreased over lactation, while fucosylation increased; secretor mothers had higher total and fucosylated HMOs.
Ma L et al., 2018, China	Glycomics	LC−MRM−MS	Not reported	Mature milk from healthy mothers.	Observed high variability in BMH concentrations, influenced by genetic and ethnic factors and secretor/blood group status
Nijman et al., 2018, USA	Glycomics	nano-LC-chip/Q-TOF MS	In-house HMOS database	Colostrum and mature milk from healthy mothers	Identified key health-related oligosaccharides (sialylated, α1,2-fucosylated, LNT), which declined from day 3 to 42.
Zhang W et al., 2019, China	Glycomics	LC–MS/MS MRM	Not reported	Mature milk from healthy mothers	Identified 12 oligosaccharides (including isomeric pairs); variations linked to donor ethnicity and timing of collection.
Yan J et al., 2019, China	Glycomics	ERLIC−MS/MS	Not reported	Transitional and mature milk from healthy mothers	Oligosaccharides declined during lactation, with neutral forms decreasing faster than sialylated ones.
Gao X et al., 2019, China	Glycomics	MALDI-TOF-MS	Not reported	Mature milk from healthy mothers	Sialylated HMOs (SHMO) reduced leukocyte adhesion; 3′-SL had antiviral effects; 2′-FL and 3′-SL improved memory and learning in infants.
Tonon M, 2019, Brazil	Glycomics	LC-MS	Not reported	Transitional and mature milk from healthy mothers and mothers with allergic disease	Higher 2′-FL in mothers of girls, higher LNH in mothers of boys; allergic mothers had higher DFpLNnH; secretor phenotype linked to allergy incidence.
Mernie E, 2019, Taiwan	Glycomics	TLC MALDI-MS	Not reported	Colostrum, transitional and mature milk from healthy mothers	Identified 25 neutral HMOs; early milk richer in large oligosaccharides; no sialylated HMOs detected.
Wang M et al., 2020, China	Glycomics	HPLC-ESI-MS	Not reported	Colostrum, transitional and mature milk from healthy mothers	Secretor phenotype influenced by ethnicity; multiparity associated with higher LNT and LNnT, lower 3′FL.
Ferreira A et al., 2020, Brazil	Glycomics	HPLC-FL	In-house HMOS database	Colostrum, transition milk and mature milk from healthy mothers	Secretor pattern more common in Latin women; multiparity linked to higher LNT and LNnT, lower 3′FL; mechanism unclear.
Siziba L, 2021, Germany	Glycomics	LC-MS2	Not reported	Mature milk from healthy mothers	Oligosaccharides decreased at 6–12 months; secretor mothers produced more; early FUT-dependent HMO exposure may be beneficial.
Siziba L, 2022, Germany	Glycomics	LC-ESI-MS	Not reported	Mature milk from healthy mothers	No association found between HMOs and atopic dermatitis at 1–2 years; identified several key HMOs.
Xun Y et al., 2022, China	Glycomics	UPLC-MS	Not reported	Mature milk from healthy mothers	Multiparous mothers had higher LNT and LNnT, lower 3FL, regardless of secretor status; explained by FUT2/FUT3 gene expression.
Vinjamuri A, 2022, USA	Glycomics	nano–HPLC–time of flight (TOF)–MS	Not reported	Mature milk in healthy mothers	HMO abundance decreased in first 6 months; α(1,2)-fucosylated species varied most; secretor mothers had higher 2′FL and LDFT, non-secretors had higher α(1,3)/α(1,4)-fucosylated HMOs.
Donatella Fortunato et al., 2003, Italy	Proteomics	MALDI-TOF-MS	PROSITE database	Colostrum from healthy mothers	Identified 107 proteins, mainly lactadherin, butyrophilin, lactoferrin, adipophilin, and carbonic anhydrase.
Gianluca Picariello et al., 2008, Italy	Proteomics	MALDI-TOF-MS	Databases from the National Center for Biotechnology Information (nrNCBI)	Colostrum from healthy mothers.	Identified 32 glycoproteins with 63 N-glycosylation sites, mainly polymeric Ig receptor, lactoferrin, IgA2 chain, serum albumin, and caseins.
Liao et al., 2011, USA	Proteomics	LC-MS/MS	Uniprot database	Colostrum, transition milk and mature milk from healthy mothers	Identified 115 proteins; early lactation showed higher expression of R-1-antitrypsin, carbonic anhydrase, chordin-like protein 2, and galectin-3 binding protein; later lactation showed higher fatty acid-binding protein, lysozyme C, and monocyte differentiation antigen.
Liao et al., 2011, USA	Proteomics	LC-MS/MS	Uniprot database	Colostrum, transition milk and mature milk from healthy mothers.	Identified 191 MFGM proteins: 21.5% involved in energy metabolism, 8.4% in growth, 19.9% in immune functions; novel proteins included polymeric immunoglobulin receptor and HLA antigens.
Liao et al., 2011, USA	Proteomics	LC-MS/MS	Not reported	Colostrum, transition milk and mature milk from healthy mothers.	Identified 82 proteins (18 specific to casein fraction) grouped into nine functional categories, highlighting immune response (28%), metabolism (22%), and cell communication (18.3%); casein fraction lacked proteins regulating nucleic acid metabolism.
Smilowitz et al., 2013, USA	Proteomics	nanoLC-Chip/TOF MS	Not reported	Mature milk from healthy mothers and mothers with gestational diabetes mellitus (GDM)	Compared to controls, GDM milk had 63.6% less sIgA, 45% more lactoferrin N-glycans, 36–72% more fucosylated and sialylated lactoferrin N-glycans, and 32–43% less sIgA and mannose, fucose, and sialic acid N-glycans.
Mandal et al., 2014, India	Proteomics	MALDI-TOF-MS	NCBI and SwissProt databases	Mature milk from healthy mothers	Identified 24 peptides with antimicrobial, antioxidant, and growth-stimulating activities; lactoferrin- and casein-derived peptides showed antibacterial, proliferative, and antioxidant effects.
Dmitry Grapov et al., 2014, USA	Proteomics	LC/MS/MS	Uniprot and Repository of Adventitious Proteins databases	Colostrum from GDM and non-GDM mothers.	Identified 27 proteins, with 10 differing between GDM and controls; GDM milk had more apolipoprotein A1, heavy chain Ig V–II region, and prostatin.
Kasper A. Hettinga et al., 2015, USA	Proteomics	LC/MS/MS	Uniprot database	Mature milk from allergic and non-allergic mothers	Identified 357 proteins in allergic mothers and 355 in non-allergic mothers; 9 were unique to allergic and 7 to non-allergic mothers.
Lingli Chen et al., 2018, USA	Proteomics	LC-MS/MS	UniProt database	Colostrum from hypothyroid and non-hypothyroid mothers	Identified 44 proteins; 29 related to energy metabolism and cell structure decreased in hypothyroid mothers, while 15 immune-related proteins increased.
Laura Di Francesco et al., 2018, Italy	Proteomics	MALDI-TOF-MS	Not reported	Colostrum and mature milk from healthy mothers	Early milk showed higher signals for peptides, proteins, and vitamins compared to mature milk, with a higher proportion of proteins <10 kDa in colostrum.
Jing Zhu et al., 2019, Netherlands	Proteomics	LC-MS/MS	UniProt database	Mature milk from healthy mothers	Identified 109 non-human peptides grouped into 36 proteins, mainly bovine caseins and β-lactoglobulin, likely from maternal diet.
Lina Zhang et al., 2019, China	Proteomics	LC-MS/MS	UniProt database	Mature milk from healthy mothers	Identified 693 proteins, most abundant were lactoferrin, serum albumin, polymeric Ig receptor, macrophage mannose receptor 1, and bile salt-activated lipase; 34 proteins varied by geography and ethnicity.
Jing Zhu et al., 2020, Netherlands	Proteomics	LC/MS/MS	Uniprot Swiss-Prot database	Colostrum, transition milk and mature milk from healthy mothers.	Identified 1,320 proteins and 2,096 peptides, adding 60 novel proteins to the human milk peptidome; 65% of peptides belonged to caseins, followed by osteopontin.
Kelly A. Dingess et al., 2021, Netherlands	Proteomics	LC-MS/MS	UniProt database, Byonic database	Transitional and mature milk from healthy mothers	Observed decreased phosphosite occupancy and O-glycosylation of β-casein during lactation, which may influence calcium binding; O-glycans localized to the β-casein C-terminal.
Vaksha Patel et al., 2021, England	Proteomics	LC-MS/MS	UniProt database	Mature milk from healthy mothers	Identified 136 previously unreported proteins, 21 linked to calcium metabolism; main proteins included serum albumin, lysozyme, lactotransferrin, and lactalbumin; FAM20A detected in all samples.
Juanjuan Guo et al., 2022, China	Proteomics	LC-MS/MS	Uniprot Swiss-Prot database, Byonic database	Colostrum and mature milk from healthy mothers and mothers with COVID-19	COVID-19 colostrum had 4.1× higher total protein, 3.9× lower caseins, and 7.2× higher whey proteins compared to controls; differences diminished by 1 month postpartum.
Marincola et al., 2012, Italy	Metabolomics	H-NRM and GC-MS	Human Metabolome Database	Transitional and mature milk from healthy mothers with preterm newborn	Observed transient changes in carbohydrate composition (increased lactose) during the first 3 weeks of life; fatty acid profile was dominated by oleic and linoleic acids; no differences between late and extreme preterm infants.
Villaseñor A, 2014, United Kingdom	Metabolomics	LC-MS and GC-MS	KEGG, METLIN, and LipidMAPS databases	Colostrum, transitional and mature milk from healthy mothers	Identified previously undescribed metabolites, including fatty esters, aldehydes, ceramides, PG and PA; also detected amino acids, organic acids, fatty acids, sugars, TCA intermediates, cholesterol, and disaccharides across lactation.
Li K et al., 2018, China	Metabolomics	UPLC-Q-TOF-MS	2002 Chinese food composition database	Colostrum, transitional milk and mature milk from healthy mothers	Identified 84 metabolites, including glycerolipids, glycerophospholipids, sphingolipids, vitamins, nucleotides, amino acids, dipeptides, steroid hormones, and others; tryptophan, histidine, and glycerolipids increased over lactation.
Gaitan A et al., 2018, USA	Metabolomics	LC-MS	Not reported	Transitional milk and mature milk from healthy mothers	Identified ARA as main metabolite; DHA, a DHEA precursor, may support infant brain and cognitive development; OEA predominated over PEA in mature milk; glycerol group accounted for > 90% of PG glycerols.
Wen L et al., 2019, China	Metabolomics	GC-MS-NMR	HMDB and NIST database	Colostrum, transitional milk and mature milk from healthy mothers and women with gestational diabetes mellitus.	Demonstrated dynamic milk metabolome over early lactation; colostrum enriched in fatty amino acids, mature milk enriched in saturated/unsaturated fatty acids and TCA intermediates; 28 metabolites differed in GDM mothers.
Isganaitis E et al., 2019, USA	Metabolomics	LC-GC-MS	Kyoto Encyclopedia of Genes and Genomes	Mature milk, from obese and healthy mothers.	Found 20 metabolites that were elevated in obese mothers’ milk; adenine positively correlated with maternal BMI and infant adiposity; suggested potential milk-mediated mechanisms for obesity transmission.
Arias-Borrego A, 2021, Spain	Metabolomics	UHPLC-MS	METLIN and HMDB database	Colostrum, from iodine-deficient mothers.	Identified 31 altered metabolites (e.g., glycerophospholipids) in iodine-deficient mothers, distinguishing them from normal, even with normal urine iodine levels; alterations may impact neurodevelopment.
Wu Y et al., 2021, China	Metabolomics	LC-MS/MS	Human Metabolome Database	Colostrum, transitional and mature milk from healthy mothers and mothers with diabetes mellitus	Identified 620 components — including keto acids, carboxylated acids, glycerophospholipids, fatty acids, organic compounds, nucleotides, and benzenoids — in healthy and diabetic mothers over the course of lactation.
Li M, 2022, China	Metabolomics	GC-TOF-MS	LECO-Fiehn Rtx5 database	Colostrum and mature milk from healthy mothers	Characterised 159 metabolites in colostrum and mature milk; 17 up-regulated and 55 down-regulated in colostrum; observed pathway differences in amino acid, lipid, inositol phosphate, glutathione, and galactose metabolism.
Zhang W, 2022, China	Metabolomics	HPLC-MS/MS	In-house MS2 database	Mature milk from healthy mothers	Identified two metabolites; PC1 associated with linoleic and α-linolenic acid derivatives; PC2 associated with phospholipids including glycerophospholipids and sphingomyelins, which may facilitate allergen absorption.
Koulman A, 2019, England	Lipidomics	LESA-MS and CID	Not reported	Transitional milk and mature milk of healthy mothers	LESA-MS revealed that mammary glands within an individual can produce distinct triacylglycerol (TG) profiles; also identified very long-chain fatty acids (C26:0 and C26:1), not previously reported in human milk.
George A et al., 2020, Australia	Lipidomics	LC-IM-MS	LIPID MAPS structure database, Australian Food Composition Database	Mature milk from healthy mothers with preterm newborn	Identified 205 TAGs, including 98 novel species; observed high levels of odd-chain fatty acids likely from diet; TAGs varied across the day, lactation stage, and between breasts; fish intake linked to DHA levels.
Xu L et al., 2020, China	Lipidomics	LC-MS/MS	Not reported	Colostrum from healthy mothers with term newborn and preterm newborn	Found significant differences in 16 lipid subclasses between term and preterm milk; preterm milk had higher phosphatidylethanolamine and phosphatidylcholine but lower diacylglycerol and ceramide; dysregulated lipids were related to metabolism, neuronal signaling, and LXR/RXR pathways.
Hewelt-Belka W et al., 2020, Poland	Lipidomics	RPLC-Q-TOF-MS	LIPID MAPS Structure Database (LMSD)	Colostrum, transitional milk and mature milk from healthy mothers	Mature milk contained 76 lipids at higher levels compared to colostrum, which had 40 lipids elevated; colostrum was richer in TGs with > 20 carbons, ether glycerophospholipids, and LC-PUFAs like eicosatetraenoic acid.
Song S et al., 2021, China	Lipidomics	LC-MS-MS	LIPID MAPS Structure Database	Colostrum, transitional milk and mature milk from healthy mothers	Phosphatidylcholine was the most abundant lipid; plasmalogens and polyunsaturated phospholipids decreased over lactation; early changes included declines in phosphatidylcholines and phosphatidylglycerols and increases in lysophosphatidylethanolamines and lysophosphatidylcholines; lipid profile stabilized after ~200 days.
Alexandre-Gouabau M et al., 2019, France	Glycomics, lipidomics, metabolomics, proteomics	LC-HR-MS	LIPID Metabolites and Pathways Strategy Database (LipidMaps), Human Metabolite Data base (HMDB), Biofluid Metabolites Database (MetLin), Milk Metabolome Database (MCDB)	Mature milk from healthy mothers	Identified biomarkers (arginine, tyrosine, hydroxybutyrate, niacinamide, choline, and lacto-N-fucopentaose I) predictive of infant weight gain; arginine, tyrosine, medium-chain fatty acids, triglycerides, and phospholipids were associated with faster growth, whereas oleic acid, plasmalogens, ceramide, and very long-chain TGs were associated with slower growth. Non-secretor mothers had very low 2′-FL, while secretors had high 2′-FL levels.
McJarrow P et al., 2019, New Zealand	Glycomic, lipidomic	HPLC-MS	Not reported	Transitional milk and mature milk from healthy mothers	Found higher concentrations of HMOs and ganglioside GD3 in transitional milk compared to mature milk, except for 3-fucosyllactose and GM3, which were higher in mature milk.

Abbreviations: HMO: Human Milk Oligosaccharides; LC-MS: liquid chromatography-mass spectrometry; MALDI-TOF-MS: matrix-assisted laser desorption/ionization time-of-flight mass spectrometry; UPLC, ultra-performance liquid chromatography; GC-MS: gas chromatography-mass spectrometry; NMR: nuclear magnetic resonance; HMDB: Human Metabolome Database; UniProt: Universal Protein Resource; FAB: fast atom bombardment; RP-HPLC, reversed-phase high-performance liquid chromatography; SEC: size-exclusion chromatography; IEC: ion-exchange chromatography; ELRIC: electrostatic repulsion hydrophilic interaction chromatography; CE-LIF: capillary electrophoresis with laser-induced fluorescence; MRM: multiple reaction monitoring; CID: collision-induced dissociation; LESA: liquid extraction surface analysis; TG: triacylglycerol; TAG: triacylglyceride; PG: glycerophosphoglycerol; PA: glycerophosphate; TCA: tricarboxylic acid; FUT: fucosyltransferase; AD: atopic dermatitis; GDM: gestational diabetes mellitus; OEA: oleoylethanolamide; PEA: palmitoylethanolamide; DHA: docosahexaenoic acid; ARA: arachidonic acid; BMH: bioactive milk human; MFGM: milk fat globule membrane.
